# Fungal spore transport by omnivorous mycophagous slug in temperate forest

**DOI:** 10.1002/ece3.8565

**Published:** 2022-02-17

**Authors:** Keiko Kitabayashi, Shumpei Kitamura, Nobuko Tuno

**Affiliations:** ^1^ Laboratory of Ecology Graduate School of Natural Science and Technology Kanazawa University Kanazawa Japan; ^2^ Ishikawa Prefectural University Nonoichi Japan

**Keywords:** basidiomycetes, endozoochory, *Meghimatium fruhstorferi*, slug, spore dispersal

## Abstract

Slugs are important consumers of fungal fruiting bodies and expected to carry their spores. In this study, we examined whether slugs (*Meghimatium fruhstorferi*) can act as effective dispersers of spores of basidiomycetes. The microscopic observation confirmed the presence of basidiospores in feces of field‐collected slugs, and the DNA metabarcoding study revealed that Ascomycota and Basidiomycota were major fungal taxa found in the feces. In Basidiomycota, the dominant order was Agaricales followed by Trichosporonales and Hymenochaetales. The laboratory experiments using *Tylopilus vinosobrunneus* showed that slugs carried a large number of spores in their digestive tracts. It was also observed that *Pleurotus*, *Armillaria*, and *Gymnopilus* spores excreted by slugs had a higher germination capacity than control spores collected from spore prints. The field experiments showed that slugs traveled 10.3 m in 5 h at most by wandering on the ground, litter layers, wood debris, and tree trunks. These results suggest that slugs could carry spores of ectomycorrhizal, saprophytic, and wood‐decaying fungi to appropriate sites for these fungi to establish colonies.

## Graphical abstract

We examined whether slugs (*Meghimatium fruhstorferi*) can act as effective dispersers of spores of basidiomycetes. Our results suggest that slugs could carry spores of ectomycorrhizal, saprophytic, and wood‐decaying fungi to appropriate sites for these fungi to establish colonies.

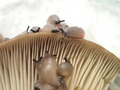

## 1 INTRODUCTION

Decomposers are indispensable components of ecosystems, and their importance is particularly high in the forest ecosystem. In a tropical forest in Malaysia, for example, 74% of the primary production is used by producers themselves for their respiration, 6% is stored as plant bodies, and most of the remaining part are directly consumed by decomposers after plant withering and death (Kira, 1983). Thus, direct biomass flows into decomposing (or saprophytic) food webs are much larger than those into food webs constituting herbivores and higher level consumers. Nevertheless, there have been only few ecological studies on organisms constituting the saprophytic food web in comparison with those on the interactions between plants and herbivores (Seibold, 2019).

In the forest ecosystem, wood debris are mainly consumed by fungi and some saprophagous and mycophagous insects (Crowson, 1984; Hammond & Lawrence, 1989; Malloch and Blackwell, 1992; Newton, 1984; Pirozynski & Hawksworth, 1988). Among them, wood‐decaying fungi play important roles in the degradation of wood debris that represent a considerable part of forest biomass. Major components of wood debris are lignin, hemicellulose, and cellulose, but many organisms do not have enzymes to digest these compounds except for wood‐decaying fungi, most of which are basidiomycetes. They digest wood materials and convert them into compounds that are digestible by insects and various other animals (Crowson, 1984; Hammond & Lawrence, 1989; Newton, 1984; Pirozynski & Hawksworth, 1988). Coarse woody debris are not decomposed by single fungi species, but by various fungi in turn before they are eventually reduced into minerals. Those wood and fungal mixtures become fresh and tasty food for certain fungivorous animals. Wood decaying is fermentation for mycophagous animals. It is believed that a complex food web starting with dead plant bodies armatured with substances that cannot be decomposed by animals is being created until the plants are finally reduced. Therefore, it would be important to understand the interactions between wood‐decaying fungi and mycophagous animals for the maintenance and regeneration of the forest ecosystem.

Wood‐decaying basidiomycetes maintain their populations by colonizing spatially scattered woody debris in the forest. It has been considered that wind plays an important role in dispersion of their spores and conidia between wood debris (Ingold, 1971; Ingold & Hudson, 1993). Insects that move between wood debris would also be efficient vectors of fungal propagules (Jacobsen, 2017; Seibold, 2019; Tuno, 1999; Vašutová et al., 2019). Well‐known examples are bark and ambrosia beetles that carry fungal hyphae or propagules of wood‐decaying fungi species‐specifically; nutritional conditions of newly colonized wood debris are largely improved for these beetles by actions of these fungi (Crowson, 1984). In contrast to such specific associations between bark and ambrosia beetles and fungi, the importance of generalist as vectors is still poorly understood. In consideration with that individual coarse wood debris are usually colonized by several fungal species (Harmon & Hua, 1991), generalist consumers feeding on wider ranges of fungal species may also play important roles in fungal dispersal. In fact, Tuno (1999) suggested that spore‐feeding beetles and flies that show specific preference to wood‐decaying fungi efficiently disperse their spores by moving between coarse wood debris. Jacobsen (2017) and Seibold (2019) also provided firm evidence that beetles and some other invertebrates play important roles in the dispersion and colonization of wood‐decaying fungi. Recently, Stephens and Rowe (2020) studied the role of generalist rodents in fungal spore dispersal networks and found that they play an underappreciated role. The role of fungivores with such a wide range of feeding habits as fungal vectors has been recognized. In these studies, however, it is not clear whether and how fungal propagules are carried by vectors to appropriate places for colony formation (Halbwachs & Bässler, 2015).

This study aims to assess a capacity of a slug species, *Meghimatium fruhstorferi*, as a vector of basidiospores. Slugs are molluscan animals assumed to play certain roles in dispersion of plant seeds and fungal propagules of various types. For example, some slugs of the genera *Arion* and *Limax* have been reported to disperse myrmecochorous seeds; these seeds are swollen by slugs because the seeds bear nutrition‐rich elaiosome that is considered to have evolved to attract ants, and swollen seeds are defecated undamaged (Türke et al., 2010, 2011). Slugs of the above two genera and some other genera such as *Agrolimax*, *Ariolimax*, and *Amalia* have also been known to feed on fruiting bodies of various fungal species (Buller, 1909, 1922; Elliott, 1922; Gain, 1891; Keller & Snell, 2002). Further, McGraw et al. (2002) reported that spores of arbuscular mycorrhizal fungi were present in feces of *Prophysaon* species and Telfer et al. (2015) found plant‐pathogenic fungal conidia in feces of *Arion vulgaris*. Voglino (1895) reported that spores of some basidiomycete species retained the germination capacity even after passing through the digestive tract of slugs. These studies indicate that slugs contribute to dissemination of fungal spores, but there have not been any more studies on this subject except the works mentioned above in our knowledge. Contributions of animals to propagule dispersal are poorly understood even in plants (Masaki, 2009), although the importance of animals and insects in pollination and seed germination has been well demonstrated (Barnea, 1991; Ollerton et al., 2011; Rader et al., 2016; Sugden, 2002). This may be because research methodology for spore and seed dispersal has not yet been established (Murray, 1988; Otani & Shibata, 2000). Recent developments in molecular biological techniques or global positioning system devices for tracking the movement of life are expected to advance research in this area (Ashley, 2010; Danks et al., 2020; Ma et al., 2016).

From the above viewpoints, we conducted field studies and laboratory experiments to evaluate the significance of *M*. *fruhstorferi* as a spore disperser. This slug occurs in mountainous forests of Taiwan and Japan (Collinge, 1901) and exploits fungal fruiting bodies, but its life histories are still poorly known (Nishi, 2015). First, we tried to determine its microhabitats by a field survey to assess their relationship to the environment in which fruiting bodies occur. We also conducted microscopic observation and the DNA metabarcoding analysis on feces of field‐captured slugs to determine the fungal species they had fed. Further, we determined the number of spores in slug’s feces and their germination capacity to assess the effectiveness of slugs as spore dispersers. Finally, we examined the travel distance and behavioral patterns of slugs with a field experimental setting to assess how far and to where slugs carry spores.

## 2 MATERIALS AND METHODS

### 2.1 Study sites

Field studies and collections of experimental individuals were carried out in a forest on the Kanazawa University campus (36°32′N, 136°42′E; 50 ~160 m in elevation) in Kanazawa City, west central Japan. This forest covers 74 ha and is dominated by deciduous broad‐leaved trees *Quercus variabilis* and *Q*. *serrata* with some stands of *Cryptomeria japonica* and *Phyllostachys edulis*. Major undergrowth plants are *Sasa palmate* and *Sasa veitchii*. In this forest, fruiting bodies of wood‐decaying fungi such as *Trametes versicolor*, *Trametes orientalis*, *Trichaptum biforme*, and *Microporus vernicipes* were frequently observed on coarse wood debris of *Q*. *variabilis* and *Q*. *serrata* from April to December and those of ectomycorrhizal fungi of the genera *Tylopilus*, *Russula*, and *Amanita* were found on the ground mainly from July to October (Tuno et al., 2020).

### 2.2 Slug’s habitats

To assess microhabitats of *Meghimatium fruhstorferi*, census was carried out along trails (a total of 2–3 km) in the study forest at a weekly interval from May to September in 2016, from June to October in 2017 and in June and July in 2018, and the number of slugs found on wood debris, fungal fruit bodies, tree trunks, and ground was recorded. The slugs found on wood debris include individuals that ate the wood‐rotting fungi that had grown on them. Wood debris are often covered with wood‐decaying fungi, and it is often difficult to determine whether they are eating or not, so they are not distinguished. Species identification of slugs followed Azuma (1995). Slugs varied in the reproductive status. In this study, those larger than 5 cm in length were assigned as adults, whereas those less than 5 cm were as juveniles. Slugs found in these censuses were collected and used in the following experiments.

### 2.3 Feeding on mushrooms and spore‐carrying capacity in the field

To assess fungus feeding of *M*. *fruhstorferi*, 34 individuals collected in the above census in 2016 were individually placed in plastic cups (350 ml) with commercial Sphagnum moss and kept at 22°C and 15‐h‐light:9‐h‐dark in an incubator. Moisture in the vials was kept relatively high by sphagnum moss. Feces excreted by slugs were collected every 24 h for 3 days and examined for the presence or absence of fungal spores with photographs taken by a digital camera (DM500, Leica Microsystems GmbH) equipped to a microscope.

### 2.4 Feeding on mushrooms in the field by amplicon analysis

For species identification of fungal species in slug’s feces, DNA metabarcoding analysis was performed. Eight *M*. *fruhstorferi* individuals collected in the census in September and October in 2017 were individually placed in plastic cups and kept under the same conditions as above to allow excretion. After 24 h, 2 g of excreted fecal samples were collected into two 1.5‐ml centrifuge tubes and stored in a freezer at −30°C. DNA metabarcoding procedures are given later.

### 2.5 Quantification of changes over time in spore excretion after eating mushrooms

To assess spore‐carrying capacity of *M*. *fruhstorferi*, 13 individuals collected in 2016 were placed in plastic cups and kept under the same conditions as above. After fasting for 3 days, they were allowed to feed dried cap of *Tylopilus vinosobrunneus* for 1 h. They were individually isolated in plastic cups with water but without food. All feces excreted were collected every 24 h until no excretion was observed at least for 24 h. Collected feces were weighed, and one g of them was suspended in water and examined for the number of spores using a blood cell calculator (Hemocytometer, Thoma) under a microscope. We counted number of spores in four squares to determine average number of spores in the chamber of the hemocytometer. We estimated the number of spores in the feces using the respective average number of spores. This density estimation method was performed according to the manual attached to the blood cell calculator.

### 2.6 Germination capacity of spores occurring in slug feces

The germination capacity of spores excreted by slugs was examined for *Pleurotus ostreatus* (Number of sporocarps = 2), *Pleurotus djamor* (*N* = 2), *Gymnopilus* sp. (*N* = 4), and *Armillaria tabescens* (*N* = 4). Fruiting bodies of these fungi and *M*. *fruhstorferi* individuals used in the experiments were collected in September 2016. Collected slugs were fasted for 3 days or longer in plastic cases at 22°C and 15‐h‐light:9‐h‐dark in an incubator and then fed with mushroom caps for 1 h and were isolated without food for sampling feces. When they started excretion, feces were collected within 2 h after excretion and suspended in sterile water. The number of spores in suspension was adjusted to 10^5^~10^6^/ml. Ten microlitre of the spore suspension was dropped on an agar plate in a Petri dish (90 mm × 20 mm), spread with a conical rod, sealed, and kept at 22°C and15‐h‐light:9‐h‐dark in a constant temperature room. Agar plates consist of a 2% agar medium supplemented with a bactericide (benomyl: 10 ppm) and an antibiotic (chloramphenicol: 100 ppm). As control, spore suspension was prepared using spores collected from spore prints of the abovementioned fruiting bodies. The suspension was adjusted for the spore density as above and inoculated on agar plates. The germination rate was monitored with randomly chosen 200 spores every 24 h for 2 or 3 days (those that extended the germination tubes were assigned to have germinated). It was difficult to observe longer because microorganisms grew on the agar plates.

### 2.7 Travel distance

Prior to field experiments to assess travel distance of slugs in the forest, we carried out a preliminary experiment to assess if slug’s activity differs between day‐time and night‐time. Ten *M*. *fruhstorferi* individuals were collected in September 2017 and kept at 22°C and 15‐h‐light: 9‐h‐dark in an incubator. Night‐time measurement was conducted from September 10 to 11; ten slugs were placed on moistened concrete floor of a parking place (ca. 30 m × 50 m) at 21:00 on September 10 and examined for their location every 30 min until 5:00 in the next morning. Day‐time measurement was carried out using the same individuals in the same way from 10:00 to 18:00 on October 14. The observers did not interfere with the movement of the slugs during the observation. The distance traveled in 30 min was assessed by measuring the minimum distance between their locations examined at a 30‐min interval, and the total distance traveled in 8 h was obtained by summing up the distances traveled in 30 min. As results, the total travel distance ranged from 6.10 to 28.30 m (mean ± SE = 16.48 ± 2.36 m) in the night‐time measurement and from 12.40 to 28.93 m (17.74 ± 3.05 m) in the day‐time measurement. There was no significant difference in the total travel distance between night‐time and day‐time (Mann–Whitney *U* test, *N* = 10, *z* = 0.843, *p* > .399).

**FIGURE 1:**
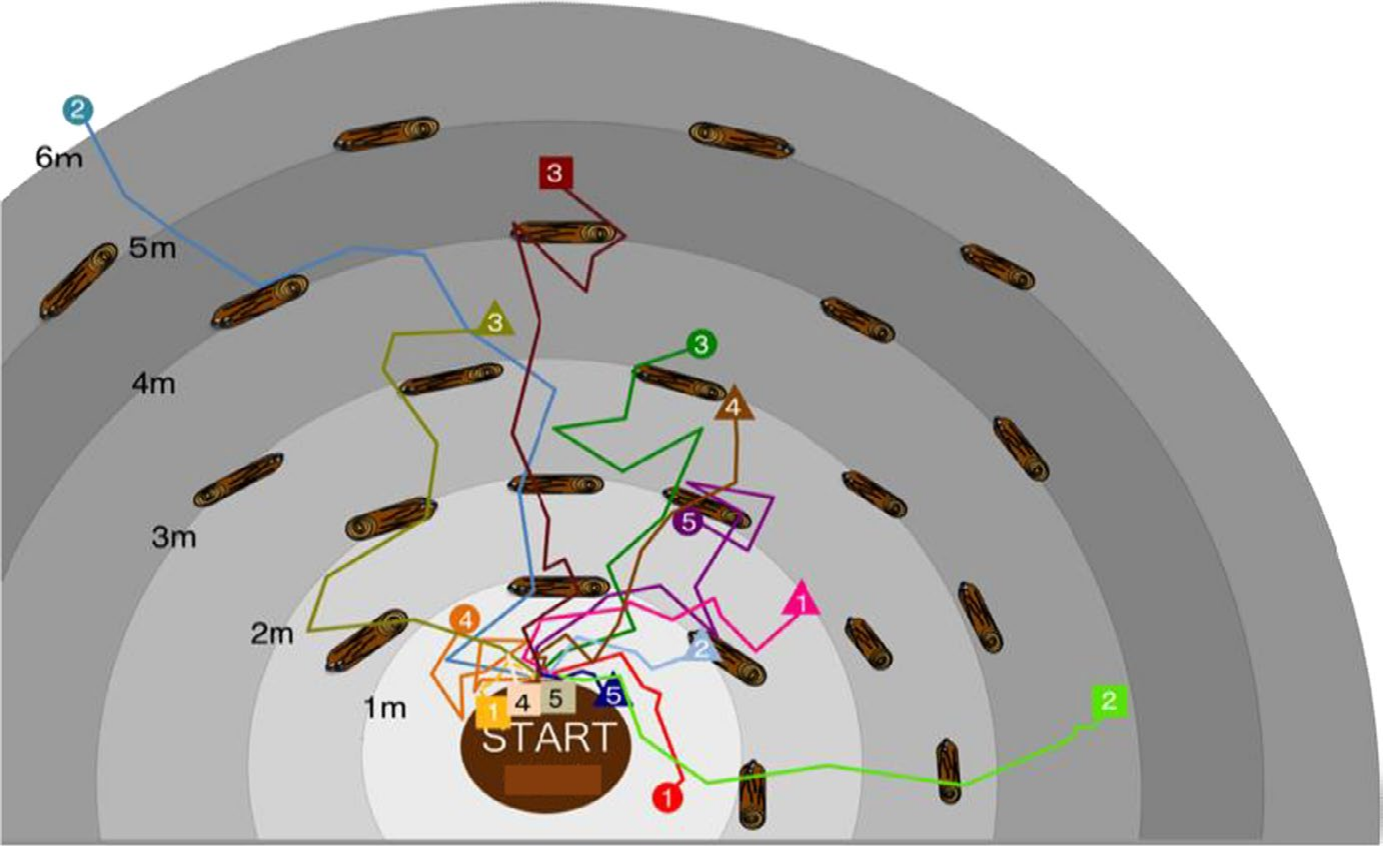
Diagram of slug travel distance survey in the field. Every 30 min after the five slugs were released from the center point, where slugs were recorded, and the points were connected to estimate their travel distance. The number is ID of the slugs. Five different slugs were observed in one trial, and it was performed three times. Symbols indicate different observation trials

Based on the above observation, we performed field experiments in day‐time, because it was difficult to observe slug movements in night in the field. We chose a flat, relatively open area in the study forest and placed 22 coarse wood debris of *Quercus* species (10~20 cm in diameter and 30~50 cm in length) radially from a *Quercus serrata* tree at an interval of approximately 1 m (Figure 1). The floor of the study area was covered by thick litter layer. Slugs used in the experiments were collected in September 2017 (their age was not known) and kept in the laboratory until experiments. Five slugs that were individually identified by their body color patterns were released at the center of the study area (i.e., at the base of the *Q*. *serrata* tree) and their locations were recorded every 30 min for 5 h. Because it was very difficult to find slugs if we once lose sight of them, at least a person kept watch on them not to lose their locations without disturbing them even if they went under fallen leaves. The distance traveled in 30 min was determined by measuring the minimum distance on the surface of ground, litter layers, tree trunks, or wood debris between their locations determined at a 30‐min interval, and the total distance traveled in 5 h was obtained by summing up the distances traveled in 30 min. We carried out the experiments three times using different slugs, November 13 (12:30 to 17:30), 14 (12:30 to 17:30), and 28 (10:35 to 15:35) in 2017. Weather conditions during the observation were cloudy and temperature was 13–15°C.

### 2.8 DNA metabarcoding by amplicon sequencing

Fecal samples were lyophilized on a VD‐250R Freeze Dryer (TAITEC Inc.) and crushed using Shake Master Neo (BMS Inc.). From the 2 g sample crushed to be uniform, a sample of about 50 mg was applied for DNA extraction using the MPure Bacterial DNA Extraction Kit (MP Bio Inc.). Library preparation was performed by a two‐step trail PCR method using ExTaq (Takara Bio Inc.); the primers for 1st PCR were 1st_ITS1‐F_KYO1 and 1st_ITS2_KYO2 (Toju et al., 2012) and the primers for 2nd PCR were 2nd‐F and 2nd‐R. In the 2‐step tailed PCR method, the first PCR is performed to amplify the target region, and the second PCR is performed for the purpose of assigning a sequence adapter and an index for sample identification. The quality of the prepared libraries was then checked using Fragment Analyzer and DNA915 Reagent Kit (Advanced Analytical Technologies Inc.).

The amplicon sequencing analysis was outsourced to Bioengineering laboratory Inc. Sequencing analysis was performed using the MiSeq Genome Sequencer under 2 × 300 bp condition. We extracted sequences whose beginning was a perfect match to the primer using fastq_barcode_splitter in the Fastx toolkit. We then used SICKLE TOOLS to remove sequences with quality values less than 20 and discarded sequences that were less than 40 bases long and their paired sequences. For lead merging, the paired‐end merge script FLASH was used to merge 320 nucleotides of the postmerge fragment length, 280 nucleotides of the lead fragment length, and ten nucleotides of the minimum overlap length. The sequences being failed to be merged were extracted, 50 bases on the 3′ side of both strands were deleted and merged again. We performed two more rounds of the same work. The reads obtained from the four merging operations were joined and analyzed in the following steps. Chimeric reads were removed from the merged reads on the basis of UNITE 97% OTU database using the USEARCH’s UCHIME algorithm. OTU creation and phylogenetic inference were performed using a workflow script from Qiime under default conditions. OTUs with less than 10 sequences were excluded from the analysis. OTUs were divided into Basidiomycota, Ascomycota, and other taxa.

## 3 RESULTS

### 3.1 Slug’s habitats

A total of 113 individuals were found in the field census from 2016 to 2018; 77 (68%) from dead woods including wood decaying fungal fruiting bodies, 22 (20%) from fungal fruiting bodies, 8 (7%) from tree trunks and 6 (5%) on the ground (Table 1). All individuals collected from fungal fruiting bodies and on the ground were adults, whereas 52.6% (*N* = 77) of individuals from dead woods and 25% (*N* = 8) of those from tree trunks were young individuals. From the results, we speculate that *M*. *fruhstorferi* breed in the caves of fallen trees and newly born slugs have lower mobility and stay around the place of birth. If these many types of spores have characteristic morphology, their higher taxa can be inferred to some extent as we reported before (Kobayashi et al., 2017). However, identification at the species level was not possible from spore shape.

**TABLE 1:** Numbers of young and adult slugs collected from dead woods, fungal fruiting bodies, ground, and tree trunk

	Young	Adult	Total
Dead woods^a^	39	38	77
Fungal fruit body	0	22	22
Ground	0	6	6
Tree trunk	2	6	8
Total	41	72	113

^a^ The number of slugs on the fruiting body of wood‐decaying fungi is included.

### 3.2 Feeding on mushrooms and spore‐carrying capacity in the field

**FIGURE 2:**
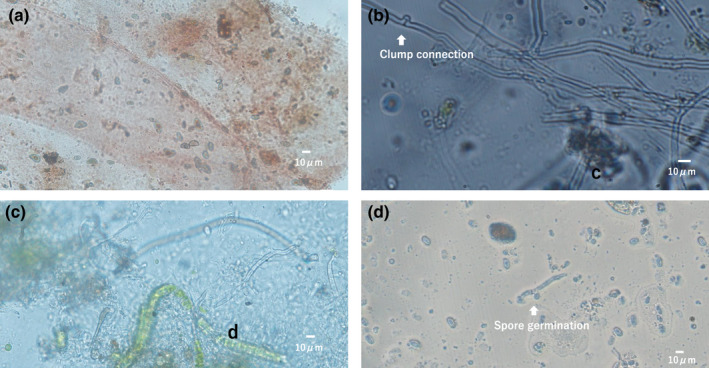
The spore and conidia‐like particles (a, b, c) and spores extending germination tubes (d) observed in slug’s faces

Spores were observed in feces of 25 (73.5%) of 34 individuals collected in September and October 2016 (Figure 2). Numerous spores were found and dikaryotic status of Basidiomycota hyphae with clamp connections were often observed (Figure 2a,b). A mixture of fungal hyphae, green algae, and cyanobacteria cells were also observed (Figure 2c). These looked like lichens but have not been confirmed. Many individuals contained spores of several types, indicating that they had fed several fungal species. Some spores in feces have already germinated (Figure 2d).

### 3.3 Feeding on mushrooms in the field by amplicon analysis

In the DNA metabarcoding study, a total of 287,174 OTU sequences were recovered from feces of eight field‐captured slugs (Table 2). Major taxa were Basidiomycota (59.1%) and Ascomycota (40.7%) in OTU read counts (Table 2). In Basidiomycota, 17 orders were detected. The dominant order was Agaricales (66.1%), followed by Trichosporonales (29.7%) and Hymenochaetales (2.9%). In Agaricales, the dominant OTUs were *Armillaria socialis* (35.7%) and *Gymnopilus patriae* (29.8%). Table 3 shows a list of dominating OTUs per individual slugs to reach 95% of the total read counts. As a result, dominating 34 OTUs in read counts are shown in Table 3. The number of OTUs does not represent the abundance of the species. However, at least within the same OTU, we think that it represents the relative quantity of whether it was consumed more or less, and Table 3 shows the number of OTU reads. This is because it would represent the difference in consumption between individual slugs. Eight slugs were found to have quite different fungal species in their excrement. For example, *Armillaria socialis* with the highest number of reads was detected in four out of eight slugs, while *Gymnopilus patriae* with the third detected in only one individual. Of the fungal species listed in Table 3, the species whose sporocarps have been confirmed to present in the field at the sampling time are marked with a plus (Table 3). We have not investigated the species of fruiting bodies in the study site by molecular method but have recorded them by morphology (Tuno et al., 2020). We have recorded that the fungal species determined as *Arimillaria socialis* by amplicon analysis was *Arimillaria tabescens* and *Gymnopilus patriae* was undescribed species of genus of *Gymnopilus*. *Trichaptum abietinum* and *Trichaptum biforme* were consistent with our identification by morphology. All of these fungal species were wood‐decaying fungi or originating from fallen trees in the field. It has also been observed that each mushroom was fed by slugs at the survey site. There are many undescribed fungal species, and the database of molecular information of fungal species is not complete, so the results of amplicon analysis are not complete, however, were almost consistent with our record of fungal species at genus level.

**TABLE 2:** Counts and read counts of fungal phylum OTUs detected in the feces of eight wild slugs captured outdoors

Phylum	OTU counts	OTU read counts	Slug ID							
			1	2	3	4	5	6	7	8
Ascomycota	752	116,895	7705	18,445	14,164	806	5884	22,313	6611	40,967
Basidiomycota	194	169,715	40,106	22,851	41,966	58,012	890	326	5360	204
Chytridiomycota	19	110	0	28	1	6	17	0	58	0
Entomophthoromycota	1	2	2	0	0	0	0	0	0	0
Glomeromycota	2	26	0	0	0	0	1	0	25	0
Mortierellomycota	16	206	25	7	42	0	51	0	52	29
Mucoromycota	13	219	29	40	3	1	27	85	10	24
Zoopagomycota	1	1	0	0	0	1	0	0	0	0
Total	998	287,174	47,867	41,371	56,176	58,826	6870	22,724	12,116	41,224

**TABLE 3:** Dominating fungal OTUs (top 34 in number of reads) in eight wild slug feces captured outdoors

Order	Species	Field obs.^a^	OTU read counts	Accumulated ratio of OTU reads	Slug ID							
					1	2	3	4	5	6	7	8
Agaricales	*Armillaria socialis*	+	60,553	0.210	19,772	3421	30,933	6427	0	0	0	0
Chaetothyriales	Herpotrichiellaceae sp.		60,047	0.418	0	5377	4788	31	290	17,187	152	32,222
Agaricales	*Gymnopilus patriae*	+	50,488	0.593	0	0	0	50,488	0	0	0	0
Trichosporonales	*Vanrija humicola*		49,038	0.763	19,914	17,901	10,255	919	0	0	0	49
Saccharomycetales	Saccharomycetales sp.		18,857	0.828	5535	8410	4860	52	0	0	0	0
Pezizomycotina ord Incertae sedis	*Alatosessilispora bibrachiata*		4557	0.844	0	0	5	0	1	1077	0	3474
Hymenochaetales	*Trichaptum abietinum*	+	4250	0.859	0	0	0	0	0	0	4250	0
Capnodiales	*Saxophila tyrrhenica*		3239	0.870	0	165	197	0	3	1251	0	1623
Pezizales	*Tuber macrosporum*		2454	0.878	884	1006	524	40	0	0	0	0
Hypocreales	*Hypomyces subiculosus*		1560	0.884	0	0	0	0	0	0	1560	0
Hypocreales	*Fusicolla aquaeductuum*		1462	0.889	0	0	0	0	1460	0	2	0
Teloschistales	*Physcia alba*		1424	0.894	0	0	0	0	0	0	1424	0
Capnodiales	Teratosphaeriaceae sp.		1249	0.898	16	227	455	0	533	0	18	0
Trichosporonales	Trichosporonaceae sp.		1206	0.902	14	618	572	0	0	0	2	0
Chaetothyriales	Herpotrichiellaceae sp.		1161	0.906	0	28	19	0	43	483	16	572
Erysiphales	*Microidium phyllanthi*		1126	0.910	0	1	0	0	0	665	6	454
Chaetothyriales	Herpotrichiellaceae sp.		1099	0.914	0	116	72	0	2	213	2	694
Hypocreales	Ophiocordycipitaceae sp.		1021	0.917	0	94	927	0	0	0	0	0
Hypocreales	Hypocreaceae sp.		983	0.921	0	0	0	0	0	0	983	0
Saccharomycetales	*Scheffersomyces stipitis*		911	0.924	0	0	0	0	0	387	0	524
Agaricales	*Simocybe sumptuosa*		778	0.927	3	0	0	0	775	0	0	0
Hymenochaetales	*Trichaptum biforme*		619	0.929	0	0	0	0	0	0	619	0
Saccharomycetales	*Hyphopichia heimii*		614	0.931	0	519	95	0	0	0	0	0
Capnodiales	Teratosphaeriaceae sp.		613	0.933	0	228	349	0	25	2	2	7
Trechisporales	Hydnodontaceae sp.	+	569	0.935	0	530	39	0	0	0	0	0
Saccharomycetales	*Candida railenensis*		521	0.937	14	412	79	0	0	0	16	0
Capnodiales	*Neotrimmatostroma excentricum*		511	0.939	0	55	123	0	6	39	13	275
Capnodiales	Teratosphaeriaceae sp.		482	0.940	0	56	40	0	372	0	14	0
Hypocreales	*Trichoderma turrialbense*		447	0.942	1	0	0	3	101	0	342	0
Tremellales	*Saitozyma podzolica*		438	0.943	161	210	51	0	0	0	16	0
Hypocreales	*Trichoderma asperellum*		431	0.945	16	70	27	29	206	46	16	21
Eurotiales	*Penicillium melinii*		372	0.948	0	4	10	0	18	0	340	0
Capnodiales	Teratosphaeriaceae sp.		366	0.949	0	74	71	0	214	0	7	0
Schizosaccharomycetales	*Schizosaccharomyces japonicus*		323	0.950	0	0	106	217	0	0	0	0

^a^ Fungal fruit bodies were present in the field when the slugs were collected.

Many other fungi that we have not confirmed the occurrence of fruiting bodies or do not form fruiting bodies have also been detected (Runnel et al., 2015). We are not able to determine where are these fungi taken up. They can be originally coming from the gastrointestinal tracts of slugs, or they can be symbiotic fungi of the lichens. The data are stored in the link of Dryad (https://datadryad.org/stash/share/IEKsT3JHCW7xivLWhKPZj4QTI5JYPFtZmrBcPZZAsDM).

### 3.4 Quantification of changes over time in spore excretion after eating mushrooms

In the experiments assessing the excreting patterns, 11 of 13 slugs fed with mushroom cap of dried *Tylopilus vinosobrunneus* for 1 h started excretion of spores within 24 h and two did within 48h. The number of spores excreted was highest on the first day [average ± SE: (9.5 ± 4.0) × 10^7^] after feeding and decreased thereafter [the seventh day, average ± SE: (2.6 ± 2.1) × 10^4^] (Table 4). The excretion lasted 2–7 days (Table 4).

**TABLE 4:** Number of spores (×10^6^) excreted by slugs first to seventh day after feeding

Slug ID	Days since feeding						
	1	2	3	4	5	6	7
1	241.040	50.680	–	–	–	–	–
2	437.580	9.000	5.880	–	–	–	–
3	–	2.200	1.680	2.880	–	–	–
4	270.335	0.810	–	0.022	–	–	–
5	112.160	5.670	0.075	–	–	–	–
6	14.260	18.300	0.000	0.045	0.002	0.000	0.006
7	5.890	0.054	–	0.160	0.000	0.000	0.098
8	8.990	30.855	–	0.001	0.000	0.000	–
9	5.400	0.003	0.002	–	0.024	0.003	–
10	22.560	6.300	0.130	0.002	–	0.000	–
11	0.000	1.520	0.000	–	0.060	0.040	–
12	8.395	21.120	0.096	–	–	0.000	0.000
13	17.640	0.293	0.000	0.000	–	0.000	0.000
Ave.	95.354	11.293	0.874	0.444	0.017	0.005	0.026
SE±	39.736	4.074	0.614	0.376	0.010	0.005	0.021

### 3.5 Germination of spores occurring in slug feces

**FIGURE 3:**
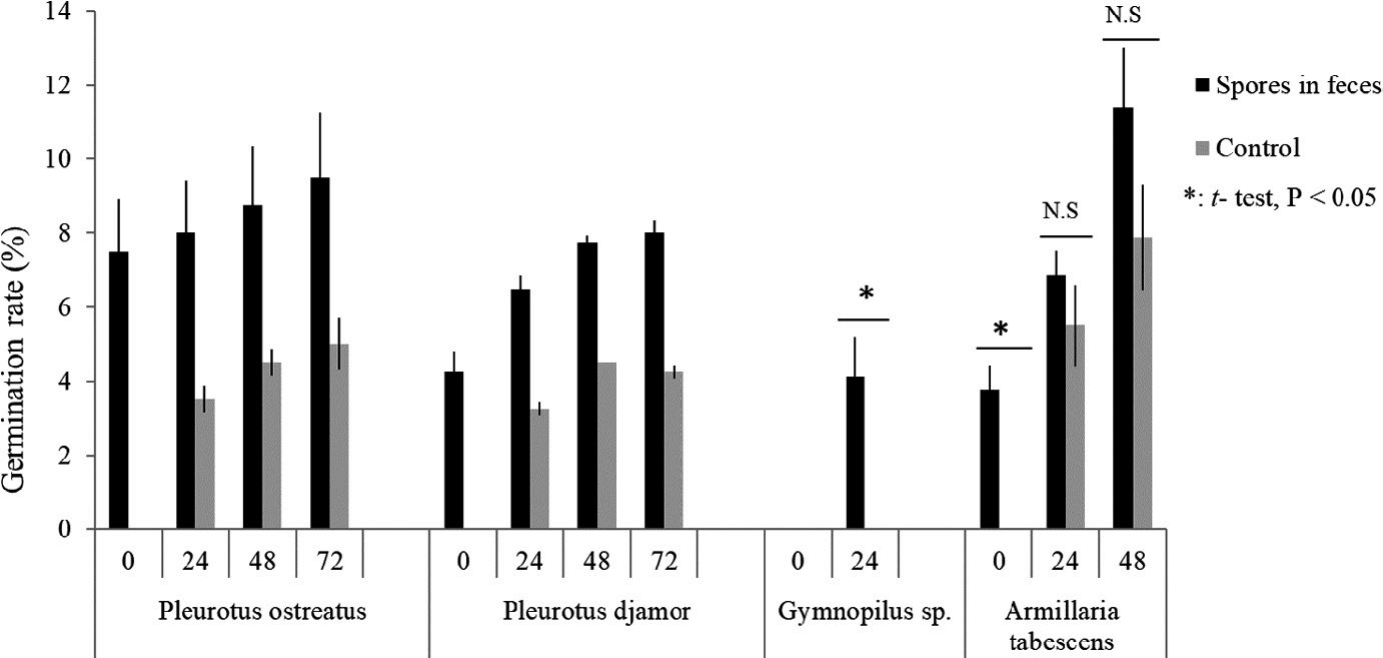
Frequency distribution of 5‐h travel distance among 15 slugs

Approximately 4%–8% of *Pleurotus ostreatus*, *P. djamor*, and *Armillaria tabescens* spores collected from feces had already germinated at the time of inoculation on agar plates (0 h in Figure 3), whereas none of control spores (i.e., those collected from spore prints) had germinated at the time of inoculation. This suggests that some of their spores had germinated in the slug’s digestive tracts or feces as we have observed in the field samples (Figure 2d). On the other hand, none of *Gymnopilus* sp. spores collected from feces had germinated at the time of inoculation (Figure 3), suggesting that its spores have longer pregermination periods than those of the above three species. Germination of spores continued at least for 72 h after inoculation (Figure 3).

### 3.6 Travel distance

**FIGURE 4:**
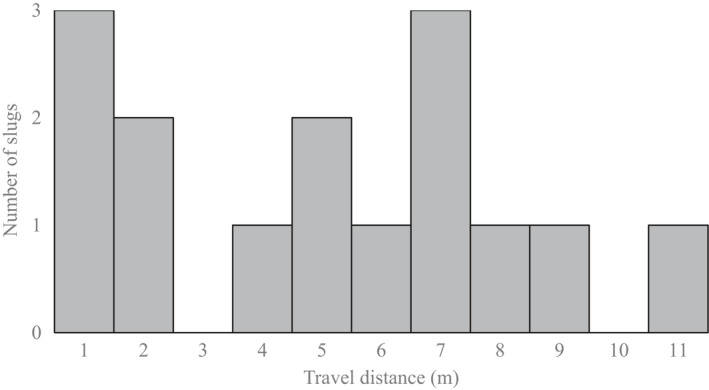
The germination rate of *Pleurotus ostreatus*, *P. djamor*, *Gymnopilus* sp., and *Armilaria tabescens* spores 0 to 72 h after collection from slug’s feces. Control spores were collected from spore prints and examined for germination 0 to 72 h after inoculation on agar plate. Results of *t* test for difference of germination rate between spores from feces and control spores are shown (*p < .05)

The frequency distribution of slug’s travel distance was bimodal rather than unimodal (Figure 4). This would be because some slugs stayed motionless for hours. If there is coarse wood debris that can be hidden, slugs stayed there for several hours. In summary, it was found that there is no such thing as periodicity in the time when the slugs do not move and the time when they travel. Among 15 individuals tested, ten moved more than 3 m in 5 h and five did less than 3 m (Figure 4). The median travel distance was 6.03 m in the former group and 0.6 m in the latter group (Table 5). The maximum travel distance was 10.35 m. In this experiment, 80% (8) of the former group and all (5) of the latter group were observed on wood debris including fallen branches or tree trunks at least once. During observation, slugs sometimes stayed motionless when they located under leaves or wood debris but never stopped movements under uncovered conditions. The number of wood debris and standing trees located per individual slugs were 2.2 in the former group and 1 in the latter group (Table 5). Our observations showed that slugs only expose their body when they travel actively but hide under wood debris or litter for hours.

**TABLE 5:** Five‐hour travel distance, the number of active and inactive slugs that located on woody debris or standing tree at least once in 5 h (A), and the number of wood debris and standing trees located per individual (B)

Slug	*N*	Travel distance (median: range)	A	B (mean ± SE)
All	15	4.95: 0.55–10.35	13 (86.67%)	1.8 ± 0.33
Active^a^	10	6.03: 3.2–10.35	8 (80%)	2.2 ± 0.44
Inactive^b^	5	0.6: 0.55–1.55	5 (100%)	1.0 ± 0.00

^a^ Group that moved more than 3 m.

^b^ Group that moved less than 3 m.

## 4 DISCUSSION

### 4.1 Exploitation of macrofungal fruiting bodies

The microscopic observation suggested the presence of basidiospores in slug’s feces and the DNA metabarcoding study revealed that feces of field‐collected slugs contained DNA fragments of Basidiomycota including the genera *Armillaria* and *Gymnopilus* (Agaricales). In agreement with these results, fruit bodies of wood‐decaying fungi are frequently observed in the study forest and fed by this slug. In the DNA metabarcoding analysis, Ascomycota fragments were also frequently detected from the slug’s feces. It is impossible to differentiate ascospores from basidiospores in our microscopic examination, although some conidium‐like cells were found. We have not observed any slugs feeding on large visual size of sporocarps of Ascomycota‐like cup fungi in the field. At present, it is not known how slugs take Ascomycota fragments. These ascomycetes fungi may be intestinal symbionts or pathobionts of slugs.

Dominance of wood‐decaying fungi in OTU read counts may be due to that the slugs used in this analysis were collected in September and October when fruiting bodies of wood‐decaying fungi were abundant in the field. We believe that the fact that slugs utilize a significantly different fungal flora for each individual means that slugs are omnivorous and are not very mobile, that is, only fungal species that can be used within their reachable range were consumed. Therefore, it seems possible to trace the mobile range of slugs by this analysis method.

### 4.2 Germination of spores excreted by slugs

The germination experiments revealed that spores of *Pleurotus* spp., *Armillaria*, and *Gymnopilus* retained the germination capacity even after passing through the slug’s digestive tract, revealing that this slug can act as dispersal vectors of fungal spores. Boch et al. (2015) also observed that moss and fern spores retained the germination capacity even after passing through the gastrointestinal tracts of large slug species, *Arion rufus*, *A. vulfaris*, and *Limax cinereoniger*. According to their study, the germination rate greatly differed by slug species. It has also been reported that seeds of some plants retained the germination capacity after passing through the digestive tracts of terrestrial shellfish (Blattmann et al., 2013; Calvino‐Cancela & Rubido‐Bará, 2012; Gervais et al., 1998; Türke et al., 2010). Spores and seeds that are often taken by animals and insects would have capacities to resist the action of digestive enzymes (Lilleskov & Bruns, 2005).

In the present experiment, the two *Pleurotus* species and *Armillaria* spores in feces had already germinated in feces or possibly in their digestive tracts. *Gymnopilus* spores did not germinate in feces, but they germinated earlier than control spores collected from sporocarps when inoculated on agar plates. Moistened environments in the digestive tracts may have enhanced germination. It is also possible that digestive enzymes in the digestive tracts may have effects. In plant seeds, it is sometimes observed that passage through the animal’s digestive tract causes physical and chemical damages to the seed coat, which increases the permeability of the seed coat to water and gases, thereby promoting germination (Traveset et al., 2008). Spores that have germinated earlier may have priority in colonization, since certain wood‐decaying fungi are competing with each other (Hiscox et al., 2015).

### 4.3 Slugs as spore disperser

For successful colony formation, spores must be transported to appropriate sites. In this respect, animals and insects could be more effective dispersal vectors than wind that carries spores over wide areas nonselectively (Galante et al., 2011). Animals and insects, on the other hand, move between favorable locations or environments for survival and reproduction and thereby could transport spores to specific sites. An example is *Muscina angustifrons* (Diptera: Muscidae); its larvae that feed ectomycorrhizal fungal fruit bodies retain a large number of spores in their digestive tracts, burrow underground for pupation, and then excrete all spores, which are expected to have larger chances to colonize seedling roots than wind‐borne spores (Kitabayashi & Tuno, 2018). In this study, slugs were observed to wander on the ground, under litters and on tree trunks and often stay on coarse wood debris, suggesting that they can carry spores of wood‐ and litter‐decaying fungi to appropriate sites for colony formation (i.e., wood debris and litters). In this respect, it is noticeable that DNA fragments of wood‐decaying fungi (*Armillaria*, *Gymnopilus*, and *Trichaptum*) were detected from slug’s feces at high frequencies. Understanding of the relationship between slugs and fungi at the species level would provide important cues to analyze the forest ecosystem.

Another important aspect of spore dispersion by animals including insects is mass transportation. It has been reported that spores of some ectomycorrhizal fungi (EMF) do not form colonies on plant roots when the density of spores is low; at least the density must be higher than 10^3^/seedling for colony formation (Rincon et al., 2001). If wood‐ and litter‐decaying fungi have also such threshold spore densities for successful colony formation, spore dispersal by animals or insects would be important for their colonization, because they carry a large number of spores (Kitabayashi & Tuno, 2018; Kobayashi et al., 2017; Tuno, 1998, 1999). Particularly, slugs are large and excrete a very large number of spores at once. According to the present study, one excrement contained 1.0 × 10^8^ spores on average. It is approximately 100–200 times larger than the number excreted by one mycophagous fly (Kobayashi et al., 2017; Tuno, 1998, 1999). Such mass transportation of spores would not be possible if transported by wind.

In addition, although data are not shown here, we also investigated the feces of spore‐feeding *Mycodrosophila* flies and *Scaphidiid* beetles at the same time as the slugs captured for amplicon analysis. We detected only their host fungus species in the spore‐feeding insects. We speculate that slugs are more omnivorous than flying insects, probably because of their low mobility. We also observed that slugs only excreted when they do not move (SK pers. obs.). It means that the ecological functions they perform can be different for host fungal species. Specifically, spore‐feeding insects may promote genetic exchange within their host fungal species, whereas slugs travel between coarse wood debris and disperse large numbers of different fungal spores those are different between slug individuals. Slugs may drastically affect the formation of the fungal flora found in dead trees. It is also noted that mucus secreted by slugs may have some roles in spore preservation and germination. This is one of the interesting future challenges.

## 5 CONCLUSION

This study revealed that basidiomycete spores fed and excreted by slugs (*M*. *fruhstorferi*) retained the germination capacity. Furthermore, slugs are assumed to carry a large number of spores to appropriate sites for colony formation, that is, under litter or on coarse wood debris. These findings suggest that slugs could be important dispersal vectors of fungal spores.

### AUTHOR CONTRIBUTION

**Keiko Kitabayashi:** Conceptualization (equal); Data curation (equal); Formal analysis (equal); Funding acquisition (equal); Investigation (equal); Methodology (equal); Project administration (equal); Resources (equal); Software (equal); Supervision (equal); Validation (equal); Visualization (equal); Writing – original draft (equal); Writing – review & editing (equal). **Shumpei Kitamura:** Conceptualization (equal); Data curation (equal); Formal analysis (equal); Funding acquisition (equal); Investigation (equal); Methodology (equal); Project administration (equal); Resources (equal); Software (equal); Supervision (equal); Validation (equal); Visualization (equal); Writing – original draft (equal); Writing – review & editing (equal). **Nobuko Tuno:** Conceptualization (equal); Data curation (equal); Formal analysis (equal); Funding acquisition (equal); Investigation (equal); Methodology (equal); Project administration (equal); Software (equal); Supervision (equal); Validation (equal); Visualization (equal); Writing – original draft (equal); Writing – review & editing (equal).

## Data Availability

The datasets generated during and/or analyzed during the current study are available from Dryad: https://doi.org/10.5061/dryad.vdncjsxvh.
